# Natural Habitat, Housing, and Restraint of Six Selected Neotropical Animals in Trinidad and Tobago with the Potential for Domestication

**DOI:** 10.1155/2020/9741762

**Published:** 2020-03-26

**Authors:** Kavita Ranjeeta Lall, Kegan Romelle Jones, Gary Wayne Garcia

**Affiliations:** The Open Tropical Forage-Animal Production Laboratory (OTF-APL), Department of Food Production (DFP), Faculty of Food and Agriculture (FFA), The University of the West Indies (UWI), St. Augustine, Trinidad and Tobago

## Abstract

This paper highlights the natural habitat, housing, and restraint needs of 6 Neotropical animals that are found in Trinidad and Tobago with the potential for domestication: agouti (*Dasyprocta leporina*), lappe/paca (*Cuniculus paca/Agouti paca*), capybara (*Hydrochoerus hydrochaeris*), manicou/opossum (*Didelphis marsupialis insularis*), collared peccary (*Tayassu tajacu/Pecari tajacu*), and red brocket deer (*Mazama americana*). The year of the earliest reference cited was 1950 and the most recent was 2018, with over 100 references being used. The average density, home range size, social group, and housing requirements were also examined as these factors would play a role in designing enclosures. A number of different physical and chemical restraint techniques were also discussed. Information from other species within the same genus was incorporated as some of the animals did not have sufficient literature.

## 1. Introduction

The world at present is having an increase in human population without enough food to feed itself. In rural communities the level of hunger and poverty is quite high. The use of indigenous Neotropical animals is a sustainable way to decease poverty and hunger in rural communities [1]. Little is known about the housing, environment, and restraint of these animals. In Trinidad, these animals are hunted as “wild meat” and are quite expensive because they are eaten as a delicacy. These animals if reared intensively can be a sustainable way for people in rural villages to increase their economic gain as well as decrease the level of poverty in these villages. These animals are native to the Neotropics and adapted to our conditions. The agouti (*D. leporina*) could be found in tropical and subtropical, moist environments and were said to be terrestrial animals. The lappe/paca (*C. paca*) was said to be a terrestrial animal that could be found near wetlands in tropical forests and they were known to burrow. The capybara (*H. hydrochaeris*) was found to be a semiaquatic animal that could usually be found in wetlands, in herds. The manicou/opossum (*D. marsupialis*) was found along wetlands and low-density woodlands and they occupied dens. The collared peccary (*T. tajacu*) could be found in diverse habitats and was described as being a social, herd animal. The red brocket deer (*M. americana*) was generally found to be a solitary animal and was known to inhabit tropical forests and wetlands with dense vegetation.

The following Neotropical animals have the potential for domestication and use to man: *Agouti* (*D. leporina*), lappe (*A. paca*), manicou (*D. marsupialis insularis*), collared peccary (*T. tajacu/P. tajacu*), and red brocket deer (*M. americana*) [2]. Hardouin and others used the phrase “mini-livestock” and defined it as any animal that is well known in its area of natural dispersion; it is not usually obtained by controlled breeding and benefits humans nutritionally or economically [1]. These animals require minimal input from man and are self-sustaining since they can be fed local feedstuff which can be collected throughout the year [3]. If these animals are to be fully utilized for meat production and conservation, then knowledge of restraint, housing, and the environment in which they dwell is critical. The purpose of this literature review is to compile information regarding the natural habitat, housing, and restraint needs of the 6 different Neotropical animals listed.

## 2. Agouti (*Dasyprocta leporina*)

### 2.1. Natural Habitat

Norris and others described agoutis in the southern Brazilian Amazon as having a bimodal activity pattern, with activity patterns being affected by the length of time forest patches have been isolated [4]. They are bimodal animals and they could be found in different types of vegetation, mainly swampy grounds and seasonally flooded areas, but they avoid areas with dense undergrowth as they preferred areas where visibility is highest, such as open areas [5]. In contrast, other authors found that they preferred a higher density of understory [6]. During the rainy season their home range was found nearer to the water line, and home ranges always included some dense forest, which provided shelter [5]. They have been described as diurnal animals that could be found in tropical and subtropical climates, in marshy woodlands near to rivers or in dry forests [7]. They were known to burrow ([7], [8]) and were described as being terrestrial animals [9, 10].

The Zoological Society of Trinidad and Tobago stated that agoutis could be found in a wide array of forests, sometimes even in gardens and crop fields [11]. The Erie Zoo stated that they could be found in tropical habitats, with a preference for areas with good undergrowth, especially near to waterways. They were found to have a wide home range that varied according to the availability of food. Males were found more in open areas as compared to females and they usually lived in pairs or small units [9].

The mean agouti density was determined in the Pinkaiti Research Station (southeastern Brazilian Amazonia) to be 31 individuals/km^2^. The average home range size was 4.5 ha, using the minimum convex polygon (MCP) approach [12]. The home range in the northern Brazilian Amazon was found to be 2.9–8.5 ha and movement was based on fruiting trees and areas of logs or treefalls. The density of adults was approximately 40 animals/km^2^ in the study site [13]. However, in Barro Colorado Island it was discovered that animals had smaller home range sizes as compared to other Brazilian studies: for male agoutis it was 2.02–4.36 ha, for females it was 0.99–2.41 ha, and for all agoutis in the study, it was 0.99–4.36 ha [14]. Other authors estimated the home range size to be 3.9–26.9 ha, with no significant differentiation between male and female home ranges, in a Brazilian Atlantic forest reserve [15] (Table 1).

The agouti density in Central Amazon, Brazil, in a continuous forest, was found to be 0.16 individuals/ha, while in 1 ha fragments the density increased and was 0.71 individuals/ha [16]. In a 4 km^2^ area there were ten social units, consisting of thirty-four individuals. A social unit was described as a family unit, with 59% of agoutis being found in units comprising two to four individuals, usually a pair with their offspring, 38% found in units comprising five to seven individuals, and only 3% lived in isolation. Females (young and adult) and adult males always lived together; however, subadult or juvenile males lived in isolation or in units with a maximum of three individuals, as adult males usually chased juveniles out. Groups inhabited areas with an average of 200 m in diameter, isolated from each other by 50 m or less. The home ranges were usually permanent, with all family members utilizing the same habitat [5]. Likewise, the San Francisco Zoo and Gardens stated that they lived in pairs or family groups [10].

### 2.2. Housing in Captivity

In Switzerland, the minimum legal measurements required for groups of up to five agoutis should be 6 m^2^ for both outdoor and indoor enclosures, and for each additional animal 1 m^2^ should be added in both outdoor and indoor enclosures. The Swiss CITES recommendations stated that for groups of up to two agoutis measurements should be 20 m^2^ for both outdoor and indoor enclosures, and for each additional animal 1 m^2^ should be added in both outdoor and indoor enclosures. At the Basel Zoo, the old enclosure size for agoutis was 50 m^2^ outdoors and about 0.5 m^2^ indoors; the new one, which catered for eight agoutis, had an outdoor measurement of 180 m^2^ and an indoor enclosure measuring 7.5 m^2^, consisting of four compartments. They were found cohabitating with birds, meerkats, and yellow mongooses, and a pool was also included (average 12.5 m^2^). At the Berne Zoo, the indoor enclosure measured 24 m^2^ for a capacity of two adults and was associated with hyacinth macaws (*Anodorhynchus hyacinthinus*). At the Zurich Zoo, the indoor enclosure measured 37 m^2^ and 16.8 m^2^ for a capacity of two adults and was associated with birds, tamarins, and marmosets [17].

**Figure 1:**
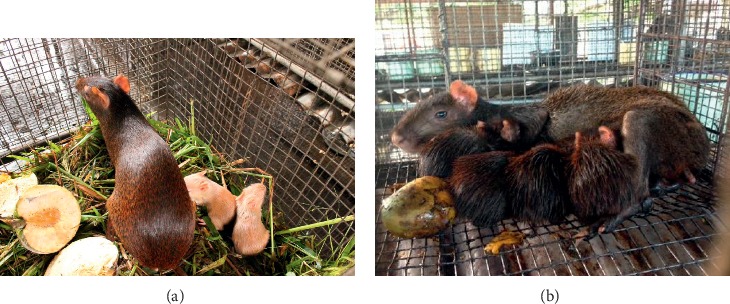
*Agouti* (*Dasyprocta leporina*/*D. aguti*) reared in captivity (in cages) at the UWI Field Station in Trinidad and Tobago (twin litter (a) and quadruplet (b)).

The Australian government stated that, in captivity, they should be housed in cages made up of metal supports and any wooden material should be secured in such a way as to prevent gnawing. Secluded areas for retreat should always be provided [18]. In captivity, caged systems and open floored systems could be used, ensuring proper ventilation (Figures 1(a) and 1(b)). It was important to have daily waste removal in addition to feeding and providing water. Pregnant animals should be separated and hiding areas for newborn and young provided (Figures 1(a) and 1(b)). The housing must have strong fences to keep animals inside, stainless steel should be used in the construction of cages so as to prevent gnawing and damage to the animals, males should be separated from juveniles and females, and there should be a male: female ratio of 1: 10. Breeding pens should measure 6′ 2″ *x* 10′ and provide hiding areas; lactation or nursing pens should measure 15′ *x* 15″ and contain leaf litter; at puberty they should be turned out to pasture in enclosures measuring 50′ *x* 60″ and mature animals should be turned out to pasture [7]. Similarly, other scientists stated that fences used in housing must be strong enough to keep agoutis inside and predators outside, and it was important to keep males separated. It was also specified that, in a 100 m^2^ pen, the maximum number of agoutis should be five [15]. Some authors stated that they were kept in pairs, in cages measuring 122 (l) × 61 (*w*) × 41 (*h*) cm with solid flooring covered with sawdust and some hay. The sides of the cage were made from metal sheets, while 5.1 × 2.5 cm mesh was used on the top and front doors. They should be supplied with hollow logs, as in the wild they would usually rest in hollow tree trunks ([19], [20, 21]).

This Neotropical rodent was described as being adapted to conditions in the tropics. The main elements for housing included isolation areas (especially for newborn and young to hide), shallow ponds (constructed using cement and measuring 15–30 cm square and 3–6 cm deep), daily supply of clean water, logs (for filing incisors), sand pits (15–30 cm square and 6–10 cm deep, filled with sand), good ventilation, proper animal visibility, sturdy gates and enclosures, and three feet high walls, with the housing as high as six feet. However, they could also be successfully kept in rabbit sized cages. Wooden material should be avoided when building the enclosures. Water supply should be done via semiautomatic drinkers; however, any containers used should be narrow enough to fit their heads only and should be made of material that could be easily cleaned and not easily destroyed by gnawing [22]. Some authors found that they could be housed with small Neotropical primates, but caution should be taken as it was risky. Some institutions have housed them with other species successfully, while there were fights and even deaths in other institutions [23].

### 2.3. Physical and Chemical Restraint

Gloves should be worn during manual restraint to prevent being bitten. Nets and transport cages could also be utilized for capture and transport [7]. These nervous animals, when very young, could be picked up using the bare hand; otherwise, nets could be used for restraint. It was noted that with constant handling, resistance decreased [19] (Table 2).

Hunters utilized their dogs to chase and trap agoutis into hollow logs, which were subsequently cut open to obtain the agoutis or the agoutis were lured out and caught in a canvas bag. They were anaesthetized using Telazol® (tiletamine hydrochloride + zolazepam) at a dose rate of 3.33 mg/kg [13]. Some authors have anaesthetized agoutis using Telazol® (tiletamine hydrochloride + zolazepam), but they used a different dose rate (0.8 mg/kg), with an onset of action of five to ten minutes [14]. Baited Tomahawk® live traps (100 × 80 × 80 cm and 81 × 23 × 23 cm) were used to capture agoutis. They anaesthetized them using a combination of ketamine (dose rate of 20 mg/kg) and xylazine (dose rate of 2 mg/kg) [15]. Physical restraint was done using hand nets. They were then anaesthetized using ketamine (dose rate of 35 mg/kg) and xylazine (dose rate of 5 mg/kg) intramuscularly and maintained using quarter dose top-ups when they were coming out of the anaesthesia [24]. Anaesthetic drugs such as ketamine hydrochloride (dose rate of 35 mg/kg) and xylazine hydrochloride (Bomazine 2%, 35 mg/ml) were used at a dose rate of 5 mg/kg to sedate animals [25] (Table 3).

## 3. Lappe/Paca (*Agouti paca*/*Cuniculus paca*)

### 3.1. Natural Habitat

Pacas were said to occupy diverse tropical habitats and could often be found in forests (ranging from deciduous to rainforests), swamps, and partially cleared grazing areas. They did not frequently disturbed habitats, but preferred low, dense tree cover. Although they often inhabited areas near to waterways, they did not live in areas with permanent water. They were known to burrow, and pairs could sometimes be found in the same burrow. Although they were described as terrestrial animals, they copulated and escaped danger in water, as they were said to be good swimmers [26]. Similarly, other authors described them as mainly nocturnal, solitary animals that lived in forested habitats close to waterways. They burrowed to a depth of 2 m and burrows contained more than one exit hole [27]. They slept in burrows, which consisted of holes, tunnels, and an internal cavity. The most commonly used enclosure had holes which would be situated around the sleeping area, with one serving as the entrance and exit to the inner cavity and the others for escape. The internal cavity had a bigger diameter than the access holes and contained litter [28]. Pacas were found near to bodies of water, while no relationship was seen between their presence and fallen fruits [29]. They preferred habitats comprising short grass savanna with dense trees and shrubs. During the wet season they avoided flooded forest areas and there was an affinity for dense understory and good canopy cover [30]. Areas with crops were positively selected, whereas secondary forests and fallows were negatively selected [31].

Male pacas were found to have larger home range sizes than females [30, 31]. In central Belize, the male's home range size was about 2.3 times greater than the female's. The home range size was 68.8–212.7 ha, with males having home ranges of 117.6–212.7 ha and females having home ranges of 68.8 ha. The mean core area (50% kernel area) was also larger for males than for females (2.7 times larger), with males having core areas of 18.1–43.2 ha and females having core areas of 10.3–13 ha. Males' home range was 87.5–204.9 ha and females' 50.7–86.7 ha [30]. The individual home range in Bolivia was 1.50–2.97 ha. Males had a somewhat larger home range size of 2.95 ha as compared to the females' mean home range size of 2.04 ha. Only one core area was determined and ranged from 0.31 to 0.54 ha [31]. Kernel analysis to determine the home ranges (95%) and core centers (50%) of pacas was used in Costa Rica. The mean home range of a juvenile male was 1.49 ha, with a mean core center of 0.22 ha. The adult female had a mean home range size of 3.44 ha, with a core center of 0.67 ha. Beck-King and von Helversen noticed a shift in home range which coincided with fruit production, which was smaller than the first, measuring 1.76 ha with a mean core center area of 0.19 ha [32].

The population density in Costa Rica was estimated using the number of burrows (93 individuals/km^2^) as well as transect census methods (67 individuals/km^2^ (Kelker's method); 70 individuals/km^2^ (King's method)) [32]. Other authors estimated the density of pacas in Bolivia and found the population density to be 6.85 individuals/km^2^, with more pacas being found in areas with dense canopies and bigger trees [33] (Table 4).

### 3.2. Housing in Captivity

**Figure 2:**
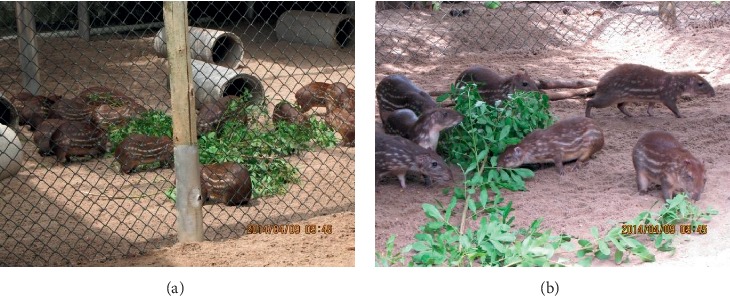
Lappe/paca (*Agouti paca/Cuniculus paca*) reared on the dirt floor with artificial concrete burrows at a 1000 head Lapp Farm in Salvador, Brazil.

The Global Federation of Animal Sanctuaries found that the minimum enclosure floor space for a pair of pacas should be 108 square feet and should contain water features to enhance the enclosures, although they are not required. The enclosures must be made using the appropriate materials, which should be maintained in good condition, and there must be appropriate drainage allowing easy cleaning [34]. The Swiss Federal Council stated that the minimum requirement for two pacas should be 8 m^2^ for the indoor enclosure, and for each additional animal 3 m^2^ should be added. In addition, the environment should allow for digging; fresh wood should be provided regularly; sleeping boxes should be included; and areas for the possibility of separating should be taken into account [35] (Figures 2(a) and 2(b)). In Switzerland, the minimum legal requirement should, for five pacas, be 8 m^2^ for the indoor enclosure, and for each additional animal 1 m^2^ should be added. The Swiss CITES recommendations stated that measurements for two pacas should be 20 m^2^ for the indoor enclosure, and for each additional animal 1 m^2^ should be added [17]. The California Natural Resources Agency recommended that the minimum enclosure floor space for one paca should be 30 square feet, with 10 square feet for each additional paca, and the height should be 5 feet. Fencing should be secured properly underground to prevent digging and subsequent escape from the enclosure. Logs for gnawing and dirt substrate should also be provided [36] (Figures 2(a) and 2(b)).

Enclosures should have concrete floors and measure 2.5–3 m. The walls should tilt inwards at the top and be constructed of two rows of cement blocks with wire at least 2 m high. The underneath of doors could have one row of blocks. Artificial burrows, with at least two entrances, should be provided for each animal in the enclosure and measure 1 (l) × 40 (*w*) × 30 (*h*) cm (Figures 2(a) and 2(b)). An additional burrow should also be provided (Figure 2). The enclosure should contain a small tub for drinking and another measuring 1 m^2^, at least 30 cm from the walls, with the floor inclined towards the tub. The lighting should be provided with a 60 W lightbulb and wires secured properly. The roof could be constructed with corrugated metal and must provide shade [37].

### 3.3. Physical and Chemical Restraint

A net, made of polypropylene material attached to a wire loop, was used to capture female pacas and they were restrained using squeeze cages made of iron [38]. Likewise, physical restraint can be obtained using hand nets [39]. Baited Tomahawk® live traps (106 × 53 × 40 cm) were reported to have captured live pacas [30]; however, some authors were not successful using these traps [40]. Pacas were captured with the aid of hunters and their dogs. When dens were discovered, all exit holes were covered except for one and was smoked out. A plastic bag was placed over the one open hole to capture the paca, which was quickly transferred, by hand, to a Havahart® type cage measuring 100 × 40 × 40 cm [31]. Utilization of trained hunting dogs was successfully used to locate pacas and capture twelve out of the sixteen seen. However, the placement of nets over the escape holes proved to be unsuccessful [40]. Another approach where burrows were excavated to attain the pacas was used [32].

A number of different chemical combinations were utilized for restraint or anaesthesia: ketamine hydrochloride (dose rate of 48.9 mg/kg) and acepromazine maleate (dose rate of 0.6 mg/kg) [30]; xylazine and ketamine at 10% intramuscularly [31]; and 10% ketamine hydrochloride intramuscularly [32]. One diazepam tablet (10 g) was given orally per paca and also midazolam maleate tablets (7.5 mg) were used, which were found to work better than the diazepam tablets [38]. Ketamine (dose rate of 25 mg/kg) and midazolam (dose rate of 0.5 mg/kg) intramuscularly were used as premedication, and isoflurane in 100% oxygen was used for induction and maintenance, via a face mask [41]. Azaperone (dose rate of 1 ml/20 kg) and promazine (dose rate of 1 ml/5 kg) were given intramuscularly [42]. Tiletamine-zolazepam (dose rate of 20 mg/kg) and xylazine (dose rate of 1 mg/kg) were given intramuscularly [43]; midazolam (dose rate of 0.5 mg/kg) and ketamine (dose rate of 25 mg/kg) were given intramuscularly [39] (Table 5).

## 4. Capybara (*Hydrochoerus hydrochaeris*)

### 4.1. Natural Habitat

In southeastern Brazil, forest cover, wetland, wetland vegetation, and water meadows influenced the occurrence of capybaras. They were mainly found in flat, open areas containing sugarcane and cultivated pasture, not in the elevated areas [44]. In the Chaco region of Paraguay, they were found to prefer primarily the Chaco forest, followed by introduced pasture, seasonally flooded wetland, open water, and lastly secondary Chaco forest. However, on the smaller scale, with respect to home range, they preferred water and then Chaco forest, followed by pasture, flooded grass area, and lastly shrub forest [45]. In Argentina, vegetation cover and “embalsados” in low-lying areas were found to be associated with their habitat. They selected habitats based on forage quality and on the presence of water, shelter, and resting sites, and they used different types of water bodies seasonally [46].

Capybaras were found to be most active during the crepuscular hours [45]. Groups usually consisted of three-four males and six females, with one male being the leader/dominant male [47]. Similarly, other authors stated that they lived in groups averaging ten adults [48]. Groups averaged five–ten individuals, making up about 80% of all groups, and comprised one dominant male, one-two adult submissive males, and four-five adult females, with the remainder being subadults or young. The male to female ratio for eighty-nine young that were either captured or captive born was 1 : 1, whereas the ratio for the adult population in the field was 1 : 3. Dominant males sometimes excluded males that reached sexual maturity from the social group. It was more common to find solitary subadult males or satellite adult males rather than females, and these individuals made up 8%. Groups consisting of males were sometimes noticed and comprised two-three members, with groups of more than four being uncommon [49].

Groups in the Llanos of Venezuela were territorial with small overlap in home ranges [50]. In Argentina, the home range size was reported to be 11.3 ha, with great overlap between groups. The core area made up an average of 22.5% of the home range, showing a 4.2% overlap, and was found to be 4.27 ha [51]. The detectability index, which was used by an observer in forested habitats in Campinas, Brazil, was 0.63 and variability in the index was due to vegetation density [52]. In Venezuela, the average population density was similar in both forested (2.06 capybaras/ha) and savanna (1.84 capybaras/ha) areas [53].

In Pantanal, Brazil, during the rainy season, due to flooding and subsequent reduction in space of a great portion of the campos, the density was the highest. The density for the frequently used habitats ranged from 5.43 to 14.82 capybaras/km^2^. The lowest densities (0.34–1.39 capybaras/km^2^) were found in the Acurizal habitat of the Pantanal of Poconé, which had no waterways or grazing areas. Groups had a core area of roughly 1 km^2^ with possibly 2 km^2^ additional shared with other groups. The most frequented areas had three aspects in common: a forest (for shelter and rest), a grazing area/campo (for foraging and daily activities), and water (source of aquatic forage and for mating), with the use of these different habitats varying seasonally [49]. Similarly, in the Llanos of Venezuela, habitat use varied seasonally. Home ranges (measured as irregular polygons) were found to be 5–16.3 ha. The average home range was 10.4 ha (ponds, 1.8 ha; Bajio, 6.6 ha; grassy banks, 0.6 ha; and bushy banks, 1.3 ha), with larger groups inhabiting larger home ranges. A feature of all home ranges was the presence of at least part of a pond/ponds and grazing area/areas [50].

In the Chaco region of Paraguay, groups consisted of up to six animals frequently found near to ponds. Home range size, using adaptive kernels, was found to be 183 ha (average 95% kernel), 64 ha (75 % kernel), and 28 ha (50% kernel). The average home range size, using MCP, was 583 ha [45]. Evaluation of different habitat types in the wetlands of Argentina found that erosion ditches had 900 capybaras/linear km of shoreline (C/LKS), protected marshes had 52.5 (C/LKS), “dirty” lagoons had 50.0 (C/LKS), “clean” protected lagoons had 30.7 (C/LKS), protected cutwaters had 27.5 C/LKS, light hunting pressure sites had 10.9 C/LKS, gallery forests under light hunting pressure had 6.3 C/LKS, and heavy hunting pressure sites had 1.0 C/LKS. The average number of capybaras/group ranged from 9.2 to 11.8 [54]. In southeastern Brazil, ecological capybara density was 124 individuals/km^2^ and was higher in the anthropogenic wetland in southeastern Brazil than in untouched habitats. However, the carrying capacity was found to be 195 individuals/km^2^ [55] (Table 6).

### 4.2. Housing in Captivity

In Switzerland, the minimum legal requirement for two capybaras should be 40 m^2^ for the outdoor enclosure and 10 m^2^ for the indoor enclosure. For each additional animal, 10 m^2^ should be added to the outdoor enclosure and 2.5 m^2^ to the indoor enclosure. They also required a pool, which should be 4 m^2^/62 m^3^. The Swiss CITES recommendations stated that, for two capybaras, the outdoor enclosure should be 150 m^2^ and the indoor enclosure 20 m^2^. For each additional animal, 10 m^2^ should be added to the outdoor enclosure and 2.5 m^2^ to the indoor enclosure. They also required a pool, which should be 2 m^3^. At the Berne Zoo, the outdoor enclosure measured 436 m^2^ and the indoor enclosure measured 15.5 m^2^ for a capacity of two capybaras, and a pool was also included. At the Zurich Zoo, the old outdoor enclosure measured 190 m^2^ and the old indoor enclosure measured 14 m^2^ for a capacity of two capybaras, and they were associated with tapir (*Tapirus terrestris*; together outdoors but separated indoors). For the new enclosures, the outdoor enclosure measured 330 m^2^ and the indoor enclosure measured 18 m^2^ (l) × 23 m^2^ (*w*) × 21 m^2^ (*h*) for a capacity of two capybaras and were associated with tapir (*Tapirus terrestris*) and anteater (*Myrmecophaga tridactyla*), which were together outdoors but separated indoors, and the pool that was included in the enclosure measured 10 m^3^ [17]. In contrast, the Swiss Federal Council stated that the minimum requirement for five capybaras should be 100 m^2^ for the outdoor enclosure and 20 m^2^ for the indoor enclosure. Each additional animal, for the outdoor enclosure, should have 10 m^2^ added and 2 m^2^ for each additional animal for the indoor enclosure. In addition, the environment should allow for digging, fresh wood should be provided regularly, and sleeping boxes should be included. Pools, with the capacity for five capybaras, should be 0.5 m deep, having a surface area of 6 m^2^ and a volume of 3 m^3^, with 1 m^2^ added for each additional capybara [35].

**Figure 3:**
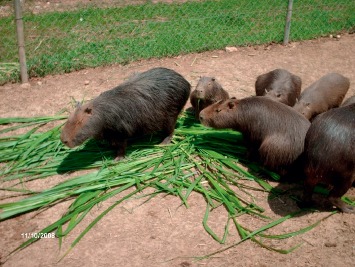
Capybara (*Hydrochoerus hydrochaeris*) housed in captivity at Marilissa Farms in Trinidad and Tobago.

The California Natural Resources Agency stated that the minimum enclosure floor space for one capybara should be 100 square feet, with 50 square feet for each additional capybara, and the height should be 5 feet. Other requirements included logs for gnawing, a pool, dirt substrate, and fencing (secured properly underground to prevent digging out from the enclosure) [36]. In comparison, the Global Federation of Animal Sanctuaries stated that the minimum enclosure floor space for one to two capybaras should be 1,600 square feet, with an additional 108 square feet for each additional animal. It was recommended that water features be 212 cubic feet, and pools should be 6 feet. Indoor enclosures should be a minimum of 15 square feet per pair, with fencing being 6 feet high. Water buckets, if used, should be high enough off the ground so that they would not defecate in the buckets [34] (Figure 3).

For a semi-intensive setting, the land should measure 2,400 m and contain lakes or ponds, trees, and/or shrubs. Enclosures should be made with mesh wire (2.5–3.0 inches) and galvanized, measuring 1.50 m in height. Water tanks should measure 4 × 5 m with a maximum depth of 0.8 m. These man-made ponds or tanks should have gently sloping sides and a ramp, at least on one side, and be made of concrete or bricks. Natural trees or artificial garages, measuring 40 m^2^, made with cement or earthenware tiles should be present to provide shelter. Two or more feeding troughs should be provided in the enclosure, measuring 0.30 × 0.30 × 1.20 m [56]. Housed groups consisted of one male with four–six females in outdoor enclosures. The enclosure was bounded by a wire mesh fence measuring 1.8 m high and enclosed an area of 120 m^2^ (sheltered area: 22 m^2^; exercise area: 98 m^2^). A drinker measuring 0.5 m^2^ and a water tank measuring 2 × 3 × 1 m deep were built into the floor of the enclosure [57]. Experimental animals could be housed individually in pens with solid floors, with drainage to the back [58].

### 4.3. Physical and Chemical Restraint

Physical restraint of capybaras was reported to have been done using a squeeze chute [58]. Snares, nets, or squeeze cages should be used for physical restraint, while chemical restraint was reported to be achieved by using a combination of a sedative that had analgesic and muscle relaxing properties, an (α_2_) Alpha_2_ agonist (such as xylazine hydrochloride, detomidine hydrochloride, or medetomidine hydrochloride), an anticholinergic agent (such as atropine sulphate), and a dissociative agent. Ketamine hydrochloride was usually used in combination with any of the aforementioned drugs [47]. Baited box-style live traps were used to catch capybaras, which were then chemically restrained using ketamine hydrochloride (dose rate of 4.7 mg/kg) and tiletamine hydrochloride/zolazepam hydrochloride (dose rate of 1.17 mg/kg) intramuscularly using a blowgun, via a 3 ml plastic dart or a 3 ml pole syringe [45]. Other chemical restraint methods included the use of ketamine (dose rate of 1.5 mg/kg) and xylazine (dose rate of 0.5 mg/kg) intramuscularly [58], and ketamine (dose rate of 5 mg/kg) and xylazine (dose rate of 0.1 mg/kg) intramuscularly via blow darts, with further anaesthesia using ketamine (dose rate of 3 mg/kg) and midazolam (0.5 mg/kg) intramuscularly [59]. The use of a blowgun was reported to deliver a fixed-ratio combination of zolazepam and tiletamine (dose rate of 3 mg/kg), morphine (dose rate of 0.3 mg/kg), and azaperone (dose rate of 1.2 mg/kg) intramuscularly [60] (Table 7).

## 5. Manicou/Opossum *(Didelphis marsupialis insularis*)

### 5.1. Natural Habitat

It was reported that *D. marsupialis* preferred low, dense woodland areas and areas near water, rather than open areas and cultivated land [61]. This was in accordance with other authors who stated that *D. marsupialis* preferred woodland habitats, and although they did not hibernate, during very cold weather they remained “holed up” for days [62]. Similarly, the California Living Museum stated that they preferred moist wetlands, brushy areas, agricultural areas, and residential areas with ample food and cover [63]. They were reported to be present in broad-leaved forests, preferring moist wetlands or areas near streams or swamps. They were observed to be nocturnal, solitary animals [64, 65], [66, 67].

In the Georgia Piedmont, the home range size for *D. virginiana* was 7.2–94.4 ha, with females having a smaller home range size as compared to males [68]. Similarly, in Wisconsin it was found that female *D. virginiana* had a smaller home range average (51 ha) as compared to males (108 ha), and there was a reduction in the home range with continuously cold weather [69]. In Missouri, the MCP method was used to determine the average home range size of *D. virginiana.* It was reported to be 37.3 ha for urban males and 18.8 ha for urban females. Core use areas were found around dens and averaged 7.47 ha for males and 4.06 ha for females [70]. In *D. marsupialis* home range overlap was variable in a ranch in central Venezuela. Females showed no overlap during the dry season but some overlap during the wet season. The home range size did not differ between wet and dry seasons. Males' home range size, during the dry season, was more than ten times larger than female home ranges, and there was overlap. Using the MCP method, the home range size was estimated at 0.30 ha for the 4 ha grid and 4.7 ha for the 20 ha grid [67]. In Costa Rica, the home range size for *D. marsupialis* was found to be 3.1–3.4 ha for females and 5.7 ha for males [71]. In northeastern Kansas the home range size of *D. marsupialis* was recorded as 5.4–164.2 acres [62].

In the Panama Canal Zone, the estimated density was recorded as 0.09–1.32 individuals/ha for *D. marsupialis* [72]. Other authors recorded similar findings in central Venezuela, having 1-2 individuals/ha for *D. marsupialis* [67]. *D. marsupialis* was found to build burrows, called dens, with multiple entrances [62, 64]. Investigators recorded that the majority of dens used by *D. virginiana* belonged to other mammals, followed by dens made in straw stacks, under small buildings, in junk heaps, in hay of an unused barn and in the hollow of a tree. All dens used never exposed them to direct sunlight [73]. Scientists examined eighty-five *D. virginiana* dens and found that the majority (90%) were located in upland pine areas, while roughly 9% were situated in upland hardwoods and one den was located near both areas. The majority of dens were located underground (with nearly 60% being stump holes), followed by ground-level dens (mainly in BlackBerry thickets and windrows) and finally arboreal dens (which were most likely originally built by squirrels) [68]. *D. marsupialis* preferred underground dens, even during the wet season [67]. They usually moved to different dens, typically every few days [68], [74] (Table 8). Dens, which could be filled with leaves or other soft material, could be found in a variety of safe, sheltered, and dry habitats and include dens built by other animals, logs, trees, stumps, crevices, and woodpiles in or under buildings [74].

### 5.2. Housing in Captivity

**Figure 4:**
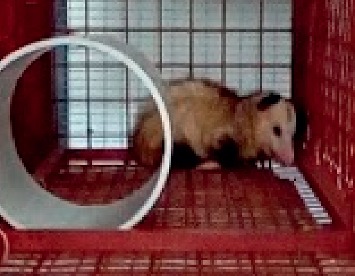
Manicou (*Didelphis marsupialis insularis*) from Trinidad and Tobago reared in a caged system.

The minimum requirement for two American opossums should be 6 m^2^ or 12 m^3^ for the indoor enclosure and 2 m^2^ for each additional animal. In addition, branches or rocks should be provided for climbing, as well as sleeping boxes [35]. One author stated that juveniles, pairs or individuals, could be housed in cages measuring 45 × 75 cm with a sloping roof (80 cm at the highest point and 35 cm at the lowest). Rabbit cages with solid floors and large ferret cages could also be used. Larger groups should be housed in enclosures measuring 30 × 50 m. Fighting could occur if the enclosures are too small. Bedding (such as newspaper) and nest boxes (30 × 45 × 40 cm) should also be provided [65]. The Blank Park Zoo housed *D. virginiana* in cages (measuring 3 × 3 × 6 m), with shelves and a place for hiding. Zoo Atlanta housed them in outdoor enclosures measuring 10 × 10 × 8 and 9 × 6 × 8, and heat lamps or frozen water bottles (depending on the temperature), nest boxes, mulch substrate, litter boxes, and climbing structures were provided [66]. Some authors have housed *D. marsupialis virginiana* in observation cages (2.5 × 1.2 × 1.2 m and 1.2 × 0.9 × 0.9 m), and nesting was facilitated by providing straw and plywood boxes, while enclosures measuring 3 × 1.5 × 1 m were used to study social behavior [75]. Enclosures for *D. marsupialis insularis* must have limited lighting and several dark areas, with climbing possibilities, and be high enough to prevent escape. Different hutches could be provided: individual hutches for breeding males, individual hutches with nesting boxes for breeding females, and grow-out hutches [64] (Figure 4).

Males housed in pairs displayed varying behaviors ranging from aggressive to regular physical contact, and this was also the case with paired females. Agnostic behavior was noticed between males and females that were not sexually responsive. Males always displayed either a sexual or submissive behavior, and after some days together, males and females seemed to tolerate each other. During the day in colder weather, they shared the same next box, but in the night females did not tolerate males, while during the summer, males and females never shared the same nest box [75].

### 5.3. Physical and Chemical Restraint

A number of different baited traps could be used to capture the *Didelphis* spp.; modified Biological Survey cat traps have been used [61]; *D. marsupialis* has been captured using live traps (46 × 15 × 15 cm) [67]; *D. aurita* has been caught using Sherman® model XLK (7.62 × 9.53 × 30.48 cm), Tomahawk® model 201 (40.64 × 12.70 × 12.70 cm), and Tomahawk® model 105 (50.80 × 17.78 × 17.78 cm) traps [76]; *D. virginiana* has been captured using box-type traps [73]; Tomahawk® single-door live traps (81 × 30 × 25 cm) have also proven to be effective [70]; and *D. virginiana* has been captured using live traps that had a double door and were collapsible [68].

**Figure 5:**
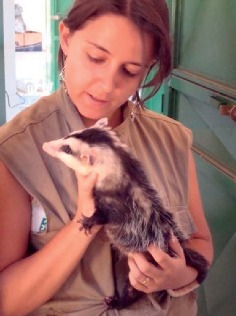
Gamba-de-Orelha-Branca (*Didelphis albiventris*) from Brazil in the Brazilia Zoo, born in captivity.

**Figure 6:**
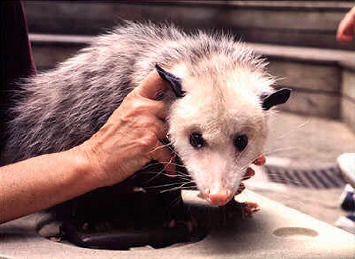
Opossum (*Didelphis virginiana*) from the Miami Metro Zoo, born in captivity.

Young opossums were reported to be held in pouches, while pet ones were handled with hands. Adults were restrained using a cat restraint bag or wrapped in a towel. Juveniles and adults were restrained using a towel over the head with one hand, while holding the base of the tail with the other hand [65]. Physical restraint was achieved by placing a towel over the animal and holding the head, or by grasping the base of the tail or grasping the base of the tail with one hand and around the neck with the other hand [77]. More feisty ones had their heads locked against a wall of the trap, using a stick, before restraining them by grasping firmly behind the head (Figures 5 and 6) [73]. Physical restraint was achieved by holding them from their tails [75], and gloves should be worn when handling [66]. *D. virginiana* was restrained using a Tomahawk® squeeze cage (50 × 27.5 × 30 cm) [78].

A number of different chemical restraint methods can be used such as ketamine hydrochloride (dose rate of 5 mg/kg) and xylazine (dose rate of 2 mg/kg) intramuscularly [70]. Pentobarbital sodium was used at a dose rate of 25 mg/kg intraperitoneally [79]. Ketamine hydrochloride was used at dose rate of 30 mg/kg intramuscularly [80], while other authors used ketamine hydrochloride at a dose rate of 25 mg/kg intramuscularly, followed by sodium pentobarbital at a dose rate of 45 mg/kg intraperitoneally [81]. Xylazine was recorded to be used at a dose rate of 0.25 mg/kg and ketamine hydrochloride at 25 mg/kg intramuscularly [82] (Table 9).

## 6. Collared Peccary (*Tayassu tajacu/Pecari tajacu*)

### 6.1. Natural Habitat

Collared peccaries have been reported to occupy a wide variety of habitats, including woodlands, tropical dry and rainforests, savannas, Gran Chaco, and deserts [83]. They have also been found in open woodland overstory and shrubland understory rather than other types, such as savannas and open or closed forests. During the day, they were mainly found in undeveloped natural areas, contrary to the night-time where they were found more in developed areas. Threatened urban peccaries' main areas of cover were shrubs. They also hid by rocks and shrubs, while grasses and trees were rarely used. Structures, such as houses, were only used during the night-time or during rain or snow storms [84]. They were also present in the semiarid, transition, and humid Chaco, and their distribution in different conditions suggested a high tolerance of human disturbance and reduced forest cover [85]. Except during the winter, they were mainly nocturnal animals [86]. Urban herds were found foraging within 250 m of housing and within 400 m of housing when bedding, while the nonurban herds were found more than 825 m from housing [87].

In Tucson Arizona, the mean herd range size of urban herds was reported to be 1.03 km^2^ (MCP method) and nonurban herds to be 1.00 km^2^ (MCP method). It was noted that individuals of the urban herds have been known to visit homes and restaurants for food [87]. Similarly, other authors found that the home range size during the day (0.2–4.3 km^2^; MCP method) was different from that during the night (0.9–6.3 km^2^; MCP method), with an overall home range size of 1.0–8.2 km^2^ in central Arizona. The core areas (0.1–1.8 km^2^; 50% MCP) were much smaller than home ranges, and while the home ranges were positively correlated to average herd size, the core areas were not. During the spring, summer, and fall, they moved more during the nights, as compared to the winter when they moved more during the early night periods. Activity level depended on the temperature and most activity took place when there was moderate temperature [84]. This was in agreement with another author who found that, in the Lower Sonoran Desert, ambient temperatures influenced movement (daily and seasonal) and the selection of shelter sites by the herds. The minimum home range was found to be 1.03–3.12 square miles in the Lower Sonoran Desert [88]. This was in disagreement with other authors who found that, in southern Texas the home range area was not influenced by the seasons. The average home range, using the minimum-area method, was 311 acres (Scott site) and 548 acres (Shaeffer site) [86]. Using the kernel 95% to determine the home range size of three herds (Herd A: maximum of 161 ha, Herd B: 243 ha, and Herd C: 157 ha) in French Guiana, it was found that, although there was seasonal variation in individual home range, mean home ranges were not seasonally affected. A positive correlation was found between home range size and fruit production. They found that the herds had a 46–81 ha overlap of home range [89].

The home range in southeastern Brazil for one herd was 308 ha (100% MCP method) or 305 ha (harmonic mean method) and for the other herd 135 ha (100% MCP method) or 123 ha (harmonic mean method) [90]. Three different groups were evaluated in the Texas Hill Country, using 95% kernel home range and 50% probability core areas, and it was found that the home range for the first group was 252 ha (core area of 39 ha), for the second group was 828 ha (core area of 157 ha), and for the third group was 427 ha (core area of 64 ha) [91]. Meanwhile, the home range size in the Tucson Mountains did not exceed 1.5 square miles and seemed not to be affected by the presence or absence of permanent water [92]. This was in agreement with other authors who determined that the home range for four herds in the Tucson Mountains ranged between 0.2 and 0.6 square miles, with home range overlap of 100–200 yards among herds. Initial herd composition varied; Herd A consisted of 4 adult females, 3 adult males, and 1 juvenile female; Herd B consisted of 2 adult females, 4 adult males, and 1 juvenile female; Herd C consisted of 7 adult females, 5 adult males, and 3 unidentified sexes; and Herd D consisted of 8 adults with unidentified sexes [93]. Individuals moved among different herds, but permanent changes in the herds did not occur frequently. A few animals were observed alone (3 males and 1 female) within the home range and they were either old or ill, but there was no evidence that they were banished from the herd. During the summer, bedding areas varied among the herds and included cool arroyos, canyons (under boulders), and under rocks. During the winter, bedding areas also varied and included mine shafts and under overhanging banks [93].

Collared peccaries were described as social animals [94], which lived in herds, with groups consisting of six to over thirty individuals [83]. This was in agreement with others who found that, in the Tucson Mountains, the average herd size was seven [92]. In a Mexican tropical forest, groups ranged from 1 to 12 individuals/herd, with 1–4 individuals/herd being the most common. Larger herds, 5–12 individuals/herd, were found in the tropical semideciduous forest, whereas smaller herds, 1–4 individuals/herd, were found in tropical deciduous forests. There was an average density of 4.9 peccaries/km^2^ [95]. Similarly, the population density in southeastern Brazil was found to be 2.8–8.9 individuals/km^2^ [90] (Table 10).

### 6.2. Housing in Captivity

**Figure 7:**
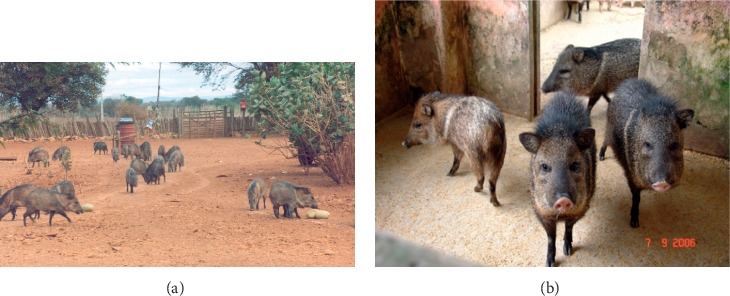
Collared peccaries being reared in an open enclosure in Bahia, Brazil (a), and on concrete floor pens in Belem, Brazil (b).

On a breeding farm in the eastern Amazon region, peccary herds were housed in different paddocks: twelve small ones (21–36 m^2^) and one large paddock (450 m^2^). In the small paddocks, the density was 0.24 individuals/m^2^ with an average space use of 4.1 m^2^/animal, while the large paddock had 25 m^2^/animal. Breeding groups consisted of 1 male : 2-3 females [96]. For enclosures, the fence must be erected in such a way as to prevent them from digging under it and escaping (Figure 7(a)). A pond or other source of water must be provided for animals to bathe and cool off [97]. In the northeastern region in Brazil a 40 ha ranch for research was built, where there was a semi-intensive production system. The paddocks consisted of the natural flooring, shrubs, trees (protected by 1.5 m high plastics or barrels), and fences (1.5 m high; constructed using wooden poles, bamboos, and living stakes). There was also electrical fencing placed 0.5 m from the wooden fencing and was 0.3–0.6 m high. There was a corral (200 m^2^) with a gate, ending with the main feeding yard on one side and a restraining area on the other side. There was also a chute with a crush box at the end, which could be alternated with a transport cage if needed [98] (Figure 7(b)). An enclosure fenced with chain link wire, set in concrete, not more than 6 feet high, and measuring 94.5 m^2^ could hold two breeding males, six breeding females, twelve growers, and twelve finishers and could be divided as further explained: The breeding males' enclosure could measure 2 × 9 m (18 m^2^) with partially paved flooring and must be covered. Maternity pens could measure 2 × 9 m (18 m^2^) with a capacity of 3 m^3^/head and include individual 1.5 × 2 m (3 m^2^) pens, two service gates, and wind break area, and these pens must be covered and paved with proper drainage. The pens could have a 20 cm high block border with chain link wire up to 2 m high. The growers' enclosure could measure 2.5 × 9 m (22.5 m^2^) with four 2 m gates and a paved and covered 2 × 3 m area. The finisher's enclosure could measure 4 × 9 m (36 m^2^), with four 3 m gates and a paved and covered 4 × 3 m area [94].

### 6.3. Physical and Chemical Restraint

A number of different baited traps were reported to have been used to capture collared peccaries: corral traps (5 × 8 m; metal posts with wire panels) and aluminium box traps (1 × 1 × 3 m) [91]; box traps alone [84]; box trap or net gun [87]; box-type deer traps, with mesh wire under traps in sandy areas [86]; foot snares [89]; box and wire panel traps, measuring 120 (l) × 90 (*h*) × 60 (*w*) cm [90]; box traps measuring 1.3 × 1.4 × 3.3 m [99]; trap made from a wooden holding crate which was lined with wire fencing (1 × 2 inch) and had a solid plywood door reinforced with wooden slats [92]; and permanent corral traps measuring 25 (*w*) × 27 (l) × 5.5 (*h*) feet, with V-mesh wire along the bottom and buried to about six inches and portable aluminium deer traps (4 (*w*) × 9 (l) × 4 (*h*) feet) [100].

A pole snare, placed around the neck and a stick (placed behind the canines) secured with rope could also be used as physical restraint [86]. Others used different physical methods for restraining such as using a wooden handling crate and a snare; young ones were not snared, but rather a canvas tarp was placed over their heads and they were restrained by sitting on them on the ground [92]. A pole snare placed behind the upper canines while another person restraining the hind legs was also another method used to physically restrain them [91], [100]. However, one author stated that snout snares were not tolerated well, so physically better restraints involved the use of permanent chutes or runways that could be blocked off to trap the animal [97]. The use of a hand net was also used as restraint for collared peccaries [101, 102].

A number of different chemical restraint methods were used such as ketamine at 20 mg/kg [90]; a 7 : 1 mixture of ketamine hydrochloride (100 mg/ml) and xylazine (20 mg/ml), which was hand-injected intramuscularly at 0.22 ml/kg [84]; ketamine hydrochloride used at a dose rate of 10 mg/kg intramuscularly via an air-dart pistol or a blowgun [89]; a capture-gun and barbless 1 ml syringe of phencyclidine hydrochloride at a dose rate of 1.0–2.3 mg/lb [88]; a 1 : 1 mg mixture of Telazol® (tiletamine hydrochloride and zolazepam hydrochloride) and xylazine at a dose rate of 2.2 mg/kg via a blowgun, jab pole, or hand syringe [99]; propofol used at a dose rate of 5 mg/kg, as a bolus intravenously, using top-ups of 1.25 mg/kg as they emerged out of anaesthesia [102], [101]; ketamine hydrochloride used at 20 mg/kg intramuscularly via a blowgun dart syringe [103, 104]; Sernylan® used at a dose rate of 0.25–0.35 mg/kg intramuscularly [93]; and Nembutal® (pentobarbital sodium) used at a dose rate of 5.4–18 mg/lb, Surital® (thiamylal sodium) at a dose rate of 2.8–6.7 mg/lb given intraperitoneally, or chloroform via a cloth over the nose [100] (Table 11).

## 7. Red Brocket Deer (*Mazama americana*)

### 7.1. Natural Habitat

One study stated that *Mazama* spp. inhabited tropical forests, with preference for dense thickets [105], while others found *Mazama* spp. most prevalent in tall evergreen forests, followed by short deciduous and flooding lowland forests [106], or in dense vegetation [107, 108]. Generally, they inhabited piedmont and riverine forests and were most active between sunset and sunrise [109]. They were active during the day and night, with two activity peaks (late afternoon and before sunrise), and they showed no habitat preference for Terra Firme or floodplain forest, being found in either [110]. These ruminants preferred moist Terra Firme forests and flood plains [111] and upland Terra Firme forests [112]. They were reported as nocturnal animals, with a bimodal activity pattern. However, in more protected areas, they were more diurnal as compared to the less protected areas, and they preferred areas of forest with low bamboo density in the understory [113]. Ultimate Ungulate [107] stated that they were diurnal, which was in contrast to other authors who found that they were nocturnal animals although they were active during the day, with the least activity around noon, which had the hottest hours [114]. They were present in dry forests, riparian forests, and marshes [115] but were also found along marshes, swamps, and streams, in moist and arid environments [108]. The red brocket deer (*M. rufa*) preferred the interior of forests and was mostly present in deciduous and gallery forests (46%), cerrado (5%), and beach (8%) [116]. *M. temama* preferred areas of intermediate plant species, dense understory, terrains with steeper slopes, and areas with vegetation less than 50 cm, with a preference for dense areas [117].

Reports showed that they were negatively affected by hunting and thus became more nocturnal in hunting areas [113]; there was a slightly higher density in persistent hunting areas, even though animals were overharvested [118]. *Mazama* spp. had a preference for low-dry forests in the nonhunting areas, while in the hunting areas, they preferred low-flooded forests [119].

They were reported to have small home ranges (1 km/0.6 miles) [107]. They stayed within 52.2 ha (MCP method) with the majority of time (75%) spent in the areas with greatest plant resources [114]. An estimated density of 1.92 animals/km^2^ was recorded in Bolivia [114], while the estimated density in southeastern Mexico was reported to be 0.90 deer/km^2^ [106]. Some scientists found that the relative abundance was 0.42 individuals/100 km in Lomerío and 6.97 individuals/100 km in Oquiriquia [120]. The population density in hunting sites (1.16 individuals/km^2^) was similar to that in nonhunting sites (1.14 individuals/km^2^) in northeastern Peru [112] (Table 12).

They were found to be mainly solitary animals [106, 108]. In a 14 ha fenced area in Argentina, *M. gouazoubira* animals were typically found solitary (74%), followed by groups of two animals (22%), three animals (3%), and four animals (0.5%). Adult bucks occupied large, nonoverlapping home ranges, while younger bucks varied throughout the home ranges. The buck population consisted of either one adult and two subadults or two adults and one subadult. They had overlapping home ranges (43%) with distinct core areas (with an overlap of only 20%). Throughout the study, one female maintained her home range, with her adult daughter overlapping 90% of her home range. Another adult female overlapped her mother's home range but had a different core area. Two juvenile females had the same home ranges as their mothers, while two juvenile males shared part of their mothers' home ranges as well as areas outside of the mothers' home ranges. Adult males had larger home ranges than adult females and juveniles, with all, except one buck, having well-defined core areas [121].

### 7.2. Housing in Captivity

**Figure 8:**
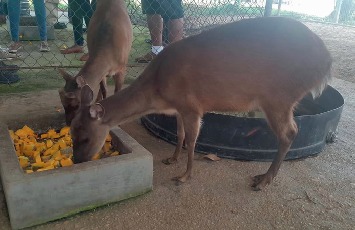
Red Brocket deer (*Mazama americana*) reared in fenced enclosure at the Sugarcane Feeds Center in Trinidad and Tobago.

The Bergen County Zoological Park successfully housed red brocket deer (*M. americana*), capybara (*H. hydrochaeris*), and greater American rhea (*Rhea americana*) together in a 10,000–20,000 square foot exhibit. The exhibit had shrubs and a large pool. Mothers and their young were kept out of the exhibit for several weeks before being reintroduced. During the day they were all exhibited together, but at night they were housed separately by species [122]. Housing conditions that were least stressful for *M. gouazoubira* consisted of keeping them in individual stalls, either all day or at night (but in outdoor exhibits during the day) [123] (Figure 8). The Phoenix Zoo successfully housed red brocket deer (*M. temama*), brown pelican (*Pelecanus occidentalis*), sandhill crane (*Grus canadensis*), screamer (*spp.*), and white pelican (*Pelecanus* spp.) together in a 1–3 acre exhibit. It was noted that the red brocket deer utilized the pond a lot. Screamers were replaced with geese when there was a conflict between them and the red brocket deer. Newborn fawns were left in the barn for four weeks before being introduced to the exhibit [122].

### 7.3. Physical and Chemical Restraint

Physical restraint was possible; however, no specific strategy was described. Authors placed their weight against or on the animals as a form of restraint [124, 125]. A number of different chemical restraint methods were implemented: Telazol® at a dose rate of 4 mg/kg with 80 mg being the total dose given [114]; a combination of ketamine (dose rate of 5 mg/kg), xylazine (dose rate of 0.3 mg/kg), and midazolam (dose rate of 0.5 mg/kg) intravenously, with isoflurane for maintenance for *M. gouazoubira* [124]; ketamine hydrochloride at a dose rate of 7 mg/kg and xylazine at a dose rate of 1 mg/kg given intramuscularly for *M. gouazoubira* and *M. nemorivaga* [126]; ketamine (dose rate of 5 mg/kg), xylazine (dose rate of 0.3 mg/kg), and diazepam (dose rate of 1 mg/kg) used intravenously and maintained using isoflurane for *M. gouazoubira* [125]; a continuous infusion of ketamine chloride at a dose rate of 10 mg/kg intravenously and xylazine hydrochloride at 1 mg/kg intramuscularly [127]; and Zoletil 50® (tiletamine/zolazepam) and Rompun 2%® (xylazine hydrochloride) intramuscularly used as light sedatives by veterinarians [128] (Table 13).

## 8. Conclusion

It was clear from this literature review that there is a need for more research with respect to these Neotropical animals, in particular, housing and restraint needs. Data from other species within the same genus were incorporated since they are closely related and thus would share similar traits.

## Figures and Tables

**Table 1:** Average density and home ranges of the agouti at different locations.

Average density (individual/km^2^)	Home range (ha)	Location	Reference
31	4.5	Southeastern Brazilian Amazon	[12]

40	2.9–8.5	Northern Brazilian Amazon	[13]

	3.9–26.9	Rio de Janeiro, Brazil	[15]

	0.99–2.41 (females) 2.02– 4.36 (males)	Barro Colorado Island, Panama	[14]

**Table 2:** Items used in the physical restraint of the agouti.

Item	Reference
Tomahawk live trap	[15]

Canvas bags	[13]

Nets	[19], [24]

Gloves worn to prevent biting; nets and cages used during transport	[7]

**Table 3:** Anaesthetics used in the chemical restraint of the agouti.

Anaesthetic	Dosage (mg/kg)	Route	Reference
Telazol® (tiletamine hydrochloride and zolazepam)	3.33		[13]

Telazol® (tiletamine hydrochloride and zolazepam)	0.8		[14]

Xylazine	2		[15]
Ketamine	20		

Ketamine hydrochloride	35	Intramuscular	[24], [25]
Xylazine hydrochloride	5		

**Table 4:** Average density and home ranges of the lappe at different locations.

Average density (individuals/km^2^)	Home range (ha)	Location	Reference
	117.6–212.7 (males) 68.8 (females)	Central Belize	[30]

6.85	2.95 (males) 2.04 (females)	Bolivia	[31], [33]

93 (no. of burrows)	1.49 (Juvenile males) 3.44 (females)	Costa Rica	[32]
67 (Kelker's method)			
70 (King's method)			

**Table 5:** Anaesthetics used in the chemical restraint of the lappe.

Anaesthetic	Dosage (mg/kg)	Route	Reference
Tiletamine-zolazepam	20	Intramuscular	[43]
Xylazine	1		

Midazolam	0.5	Intramuscular	[39], [41]
Ketamine	25		

Ketamine hydrochloride	48.9		[30]
Acepromazine maleate	0.6		

Xylazine and ketamine		Intramuscular	[31]

Ketamine hydrochloride		Intramuscular	[32]

Diazepam		Oral	[38]
Midazolam maleate		Oral	

**Table 6:** Average density and home ranges of the capybara at different locations.

Average density	Home range (ha)	Location	Reference
2.06 capybaras/ha (forested area)		Venezuela	[53]
1.84 capybaras/ha (savanna)			

5.43–14.82 capybaras/km^2^ (campos)		Pantanal, Brazil	[49]
0.34–1.39 capybaras/km^2^ (Acurizal)			

	11.3	Argentina	[51]

	5–16.3	Llanos, Venezuela	[50]

	583	Chaco, Paraguay	[45]

900 C/LKS (wetlands)		Argentina	[54]
52.5 C/LKS (protected marshes)			
50 C/LKS (dirty lagoons)			
30.7 C/LKS (clean lagoon)			
27.5 C/LKS (protected cutwater)			
10.9 C/LKS (light hunting pressure)			
6.3 C/LKS (gallery forest under light hunting pressure)			
1.0 C/LKS (heavy hunting pressure)			

195 individuals/km^2^		Southeastern Brazil	[55]

**Table 7:** Anaesthetics used in the chemical restraint of the capybara.

Anaesthetic	Dosage (mg/kg)	Route	References
Ketamine hydrochloride	4.7	Intramuscular (via a blowgun)	[45]
Tiletamine hydrochloride/zolazepam hydrochloride	1.17		

Ketamine	1.5	Intramuscular	[58]
Xylazine	0.5		

Ketamine	5.0	Intramuscular (via blow darts)	[59]
Xylazine	0.1		

Zolazepam and tiletamine	3.0	Intramuscular (via a blowgun)	[60]
Morphine	0.3		
Azaperone	1.2		

**Table 8:** Average density and home ranges of the manicou at different locations.

Average density (individuals/ha)	Home range (ha)	Location	Reference
0.09–1.32		Panama Canal Zone	[72]

1–2	0.30	Central Venezuela	[67]
	4.7		

	3.1–3.4 (female) 5.7 (male)	Costa Rica	[71]


	2.18–66.45	Northeastern Kansas	[62]

	7.2–94.4	Georgia Piedmont	[68]

	51 (female) 108 (male)	Wisconsin	[69]


	18.8 (female) 37.3 (male)	Missouri	[70]


**Table 9:** Anaesthetics used in the chemical restraint of the manicou

Anaesthetic	Dosage (mg/kg)	Route	Reference
Ketamine hydrochloride	5	Intramuscular	[70]
Xylazine	2		

Pentobarbital sodium	25	Intraperitoneal	[79]

Ketamine hydrochloride	30	Intramuscular	[80]

Ketamine hydrochloride	25	Intramuscular	[81]
Sodium pentobarbital	45	Intraperitoneal	

Xylazine	0.25	Intramuscular	[82]
Ketamine hydrochloride	25		

**Table 10:** Average density and home ranges of the collared peccary at different locations.

Average density (individuals/km^2^)	Home range	Location	Reference
4.9		Mexico	[95]

2.8–8.9	308 ha	Southeastern Brazil	[90]
	135 ha		

	1.03 km^2^ (urban herds)	Tucson, Arizona	[87]
	1.00 km^2^ (nonurban herds)		

	0.2–4.3 km^2^ (day)	Central Arizona	[84]
	0.9–6.3 km^2^ (night)		
	1.0–8.2 km^2^ (overall)		

	1.03–3.12 square miles	Lower Sonoran desert	[88]

	311 acres (Scott site)	Southern Texas	[86]
	548 acres (Shaeffer site)		

**Table 11:** Anaesthetics used in the chemical restraint of the collared peccary.

Anaesthetic	Dosage (mg/kg)	Route	Reference
Ketamine hydrochloride	10	Intramuscular (via an air-dart pistol or a blowgun)	[89]

Phencyclidine hydrochloride	2.2–5.07	Via a capture-gun and barbless 1 ml syringe	[88]

Ketamine	20		[90]

Telazol® (tiletamine hydrochloride-zolazepam hydrochloride)	2.2	Intramuscular (via a blowgun, jab pole, or hand syringe)	[99]
Xylazine	2.2		

Propofol	5.0 (bolus)	Intravenous	[102], [101]
	1.25 (top-up)		

Ketamine hydrochloride	20	Intramuscular (via a blowgun dart syringe)	[103], [104]

Sernylan®	0.25–0.35	Intramuscular	[93]

Nembutal® (pentobarbital sodium)	12–40	Intraperitoneal	[100]
Surital® (thiamylal sodium)	6.2–14.8	Intraperitoneal	
Chloroform		Inhalation	

**Table 12:** Average density and home ranges of the red brocket deer at different locations.

Average density (individuals/km^2^)	Home range (km)	Location	Reference
	1		[107]

0.90		Southeastern Mexico	[106]

1.16 (hunting sites)		Northeastern Peru	[112]
1.14 (nonhunting sites)			

1.92		Bolivia	[114]

0.42 individuals/100 km		Lomerío	[120]
6.97 individuals/100 km		Oquiriquia	

**Table 13:** Anaesthetics used in the chemical restraint of the red brocket deer.

Anaesthetic	Dosage (mg/kg)	Route	Reference
Telazol®	4		[114]

Ketamine	5	Intravenous	[124]
Xylazine	0.3		
Midazolam	0.5		

Ketamine hydrochloride	7	Intramuscular	[126]
Xylazine	1		

Ketamine	5	Intravenous	[125]
Xylazine	0.3		
Diazepam	1		

Ketamine chloride	10	Intravenous	[127]
Xylazine hydrochloride	1	Intramuscular	
